# *p*-Cresyl sulfate predicts clinical outcomes in sustained peritoneal dialysis: a 5-year follow-up cohort study and meta-analysis

**DOI:** 10.1080/0886022X.2022.2136528

**Published:** 2022-10-24

**Authors:** Zewei Chen, Jing Xu, Xiaohong Xing, Cheng Xue, Xiaoling Luo, Shouhong Gao, Zhiguo Mao

**Affiliations:** a Kidney Institute, Department of Nephrology, Shanghai Changzheng Hospital, Second Military Medical University, Shanghai, China; b Department of Pharmacy, Shanghai Changzheng Hospital, Second Military Medical University, Shanghai, China

**Keywords:** *p*-Cresyl sulfate, peritoneal dialysis, peritoneal dialysis failure, cardiovascular disease, peritoneal dialysis-associated peritonitis

## Abstract

**Background:**

The impact of *p*-cresyl sulfate (PCS) and indoxyl sulfate (IS) on the prognosis of patients with uremia remains controversial. We performed a prospective study on peritoneal dialysis (PD) to investigate the relationship between PCS or IS levels with clinical outcomes.

**Methods:**

This prospective cohort study investigated the association of serum PCS and IS with clinical outcomes in patients undertaking PD. We performed a correlations analysis to explore the influencing factors of PCS an IS. Meta-analysis was conducted to objectively evaluate the prognostic effects of PCS and IS on different stages of CKD patients.

**Results:**

A total of 127 patients were enrolled consecutively and followed with an average period of 51.3 months. Multivariate Cox regression showed that serum total PCS not only contributed to the occurrence of PD failure event (HR: 1.05, 95% CI = 1.02 to 1.07, *p* < 0.001), but also increased the risk of cardiovascular event (HR: 1.08, 95% CI = 1.04 to 1.13, *p* < 0.001) and PD-associated peritonitis (HR: 1.04, 95% CI = 1.02 to 1.08, *p* = 0.001). Dividing the total PCS level by 18.99 mg/L, which was calculated from the best cutoff value of the ROC curve, patients with total PCS higher than 18.99 mg/L had worse prognosis. Meta-analysis confirmed its value in cardiovascular event in PD.

**Conclusion:**

The serum total PCS concentration was a detrimental factor for higher PD failure event, cardiovascular event, and PD-associated peritonitis. It could be used as an innovative marker in predicting poor clinical outcome in PD.

## Introduction

Impairment of renal function results in the retention of large numbers of compounds, as known as uremic toxins or uremic retention solutes, which normally can be excreted to the urine [1–5]. In 2012, the European Uremic Toxin Work listed at least 88 retained uremic solutes including small-molecular-weight compounds, large-molecular-weight compounds, and protein-bound uremic toxins (PBUTs) as well [1,2]. Due to its high affinity for serum protein, PBUTs cannot be removed efficiently by hemodialysis (HD) or peritoneal dialysis (PD) [6,7].

Among the various members of PBUTs, *p*-cresyl sulfate (PCS) and Indoxyl sulfate (IS) are two major components [1,8]. Both PCS and IS originate from the bacterial protein fermentation in the intestine [3]. Colonic microbiota degrades tryptophan to indole and its further hydroxylation results in IS; in parallel, degradation of tyrosine ultimately gives rise to PCS. In dialysis patients, serum concentrations of PCS and IS were ∼54 and 17 times higher, respectively, compared to that of the healthy populations [2,4].

In recent years, PBUTs have gained more and more attention as a potential hazard leading to worse prognosis, however, the prognostic effect of PCS or IS on different stages of CKD patients still remains debatable. There were reports about high PCS levels that increased the risk of cardiovascular diseases in pre-dialysis CKD [9,10] and all-cause mortality in HD patients [11,12]; on the other hand, others indicated that IS was more responsible for the higher rate of all-cause mortality in HD patients [13]. However, current studies exploring the prognostic impact of PCS or IS were mainly conducted among pre-dialysis CKD or HD population, the information about relationship between PCS or IS and clinical outcome in PD was scarce. PD has been unprecedented spread in the past decade with its low invasive, cost-effective and physiological toxin removal [14,15]. Although several studies tried to decrease PBUTs level in PD, its long-term benefit on patients’ prognosis still needs further evidence. Thus, we performed a prospective study to investigate the influence of serum PCS and IS levels on clinical outcomes of patients undertaking PD. Moreover, meta-analysis was conducted to objectively evaluate the prognostic effects of PCS and IS on different stages of CKD patients.

## Methods

### Study population

The prospective study was conducted using the PD Information System of Changzheng Hospital, Shanghai (www.miraclamedicle.com:1613). We recruited 127 patients with PD starting from January 2012. Patients aged > 18 but < 75 years old undertaking continuous ambulatory peritoneal dialysis (CAPD) or automatic peritoneal dialysis (APD) longer than 3 months were eligible for inclusion. Patients were excluded when they were having (a) peritonitis in the past 1 month; (b) acute infection and cardiovascular event in the past 3 months; (c) active stage of autoimmune diseases; (d) having suffering malignancy. The study was performed in accordance with the principles of the Declaration of Helsinki and approved by the Ethics Committee of Changzheng Hospital (ChiCTR-IOR-14005541).

### Baseline evaluation and biochemical measurement

Data on baseline demographics and biochemistry examination were obtained after signing consent for all patients. The following tests were performed: hemoglobin (g/L), Albumin (g/L), Creatinine (µmol/L), Urea nitrogen (mmol/L), Uric acid (µmol/L), Calcium (mmol/L), Phosphate (mmol/L), Bicarbonate (mmol/L), and PTH (pg/mL). Kt/V and creatinine clearance rate (Ccr) for PD were calculated at the time of the first peritoneal equilibration test after signing consent for all patients. All serum IS and PCS were measured two times to obtain an average value. The first measurement of serum PCS and IS was performed at the beginning of recruitment, and the second was performed at the third month after the first. The metabolic syndrome (MS) is defined consistently as previously published literature [16].

#### PCS and IS examination

The serum concentrations of total and free PCS and IS were determined by HPLC/MS/MS method as previously described [17]. Serum samples were prepared by centrifuge as required and HPLC/MS/MS analysis was performed on an Agilent 1200 series HPLC interfaced to an Agilent 6410 A triple-quadrupole mass spectrometer equipped with an ESI source (Agilent Inc, MA, USA). The separation was carried out on an Agilent Zorbax SB-C18 column with the column temperature maintained at 30 °C. The mobile phase consisted of a mixture of acetonitrile and 10 mM ammonium acetate buffer (10:90, v/v) using an isocratic elution at a flow rate of 0.3 mL/min. A 5-µL aliquot of sample solution was injected and analyzing time of each injection was 5 min. Quantitation was performed using electrospray in the negative mode with the spray voltage was set at 4000 V. The linearity ranged from 500 to 10,000 ng/mL for IS (*r* > 0.99) and 50 to 10,000 ng/mL for PCS (*r* > 0.99). The limit of detection was 500 ng/mL for IS and 50 ng/mL for PCS. Relative standard deviation (SD) of intra- and inter-day precision was with ± 15%.

### Clinical endpoint evaluation

Patients were followed-up and visited the PD clinic at 3–6-month intervals with clinical and biochemical data recorded. Clinical endpoints were prospectively collected. The patient was assumed to be lost to follow-up starting from the date of the last actual visit. The major endpoint was PD failure event, which was defined as a composite of switching to HD or all-cause mortality during the study period. The secondary endpoints were cardiovascular event and time-to-first PD-associated peritonitis. Cardiovascular event was defined as patients with any one of the following including death from cardiac causes, myocardial ischemia, nonfatal myocardial infarction, ischemia stroke, or new onset of peripheral vascular disease, whichever occurred first [7,9]. PD-associated peritonitis was diagnosed if participants meet at least two or the following criteria: (i) abdominal pain and/or opacity of dialysis effluent; (ii) dialysis effluent with white blood cell count more than 100 cells/mm,^3^ with neutrophils leukocytes 50% or more on a 2-h dwell sample; and (iii) positive effluent Gram staining [18]. The earliest occurring event per subject was included in the analysis.

### Literature review and data extraction of meta-analysis

Meta-analysis was performed to objectively evaluate the prognostic impact of PCS and IS on different stages of CKD, including pre-dialysis and dialysis patients. We searched the PubMed, EMBASE, Cochrane, and Web of Science databases with terms including ‘uremic toxins’, ‘protein-bound toxins’ or ‘PCS’ combined with the terms ‘chronic kidney disease’ or ‘CKD’ or ‘end stage renal disease’ or ‘ESRD’ or ‘dialysis’. The systematic literature review was screened with data extracted meanwhile independently by Zewei Chen and Jing Xu.

### Statistical analysis

Continuous variables were expressed as mean ± SD for normally distributed variables or median (interquartile range) otherwise. Categorical variables are expressed as frequencies (percentage). Comparisons of continuous and categorical data for patients in different groups were performed by independent samples *t* tests. Spearman’s rank correlation or Pearson’s correlation coefficient were used to estimate the relationships between serum PCS or IS levels and selected variables. Cox regression model was used to analyze the relationship between independent variables and clinical outcomes, and reported by Hazard ratio (HRs) with 95% confidence intervals (CIs). The ROC curve was used to estimate the best cutoff serum concentrations of total-PCS of PD failure event. The Kaplan-Meier method (factors were compared using the log-rank test) was used to estimate cumulative survival rate of time to clinical endpoints in PD patients with total PCS above and below the best cutoff point. For the meta-analysis, adjusted point estimates from each study were consolidated by the generic inverse variance approach, which designated the weight of each study based on its variance. Forest plots for outcomes were generated with 95% CIs. Given the possibility of between-study variance, we adopted a random-effects model rather than a fixed-effect model. Date was analyzed using the SPSS 26.0 software and Review Manager 5.3. The authors had full access to the data and take responsibility for its integrity. *p* < 0.05 was considered statistically significant.

## Results

### Patient demographics

A total of 127 patients undertaking sustained PD were recruited consecutively from January 2012 to February 2015 and ended on February, 2020 with an average age of 44.7 ± 13.9 years old, and included 77 males (60.6%). The etiology of ESRDs was chronic glomerulonephritis, nephrotic syndrome, IgA nephropathy, diabetic nephropathy, hypertensive nephropathy, or polycystic kidney disease. The demographic and clinical characteristics were shown in Table 1. Median level of serum total and free PCS was 17.8 (7.2, 25.2), 1.44 (0.51, 1.79) mg/L, respectively; and that of total and free IS was 23.6 (11.1, 32.6), 1.90 (0.65, 2.18) mg/L, respectively.

**Table 1.** Baseline characteristics of the study patients.

Variables	PD patients (*n* = 127)
Age (years)	44.7 ± 13.9
Male (%)	77 (60.6)
PD duration (months)	15.6 ± 3.9
MS (%)	11 (8.7)
Hypertension (%)	114 (89.8)
Diabetes mellitus (%)	14 (11.0)
Total-IS (mg/L)	23.6 (11.1, 32.6)
Free-IS (mg/L)	1.90 (0.65, 2.18)
Total-PCS (mg/L)	17.8 (7.2, 25.2)
Free-PCS (mg/L)	1.44 (0.51, 1.79)
Peritoneal transport type	
Low (%)	3 (2.4)
Low average (%)	42 (33.1)
High average (%)	73 (57.5)
High (%)	9 (7.1)
D/Pcr	0.67 ± 0.09
Peritoneal Kt/V	1.35 (1.00, 1.54)
Residual renal Kt/V	0.69 (0.18, 0.92)
Peritoneal Ccr	37.8 (30.9, 43.3)
Residual renal Ccr	35.3 (13.8, 46.0)
nPCR (g/kg/day)	0.85 ± 0.19
Hemoglobin (g/L)	97.9 ± 18.6
Albumin (g/L)	35.2 ± 4.9
Creatinine (µmol/L)	929.9 ± 319.3
Uric acid (µmol/L)	453.1 ± 94.9
Calcium (mmol/L)	2.15 ± 0.30
Phosphate (mmol/L)	1.91 ± 0.58
Bicarbonate (mmol/L)	21.31 ± 4.12
PTH (pg/mL)	314.2 (148.3, 351.8)

Ccr: creatinine clearance rate; D/Pcr: dialysate/plasma creatinine ratio; IS: indoxyl sulfate; MS: metabolic syndrome; nPCR: normalized protein catabolic rate; PCS: *p*-cresol sulfate; PD: peritoneal dialysis; PTH: parathyroid hormone.

### Clinical outcomes

#### Primary and secondary endpoints

**Figure 1.**
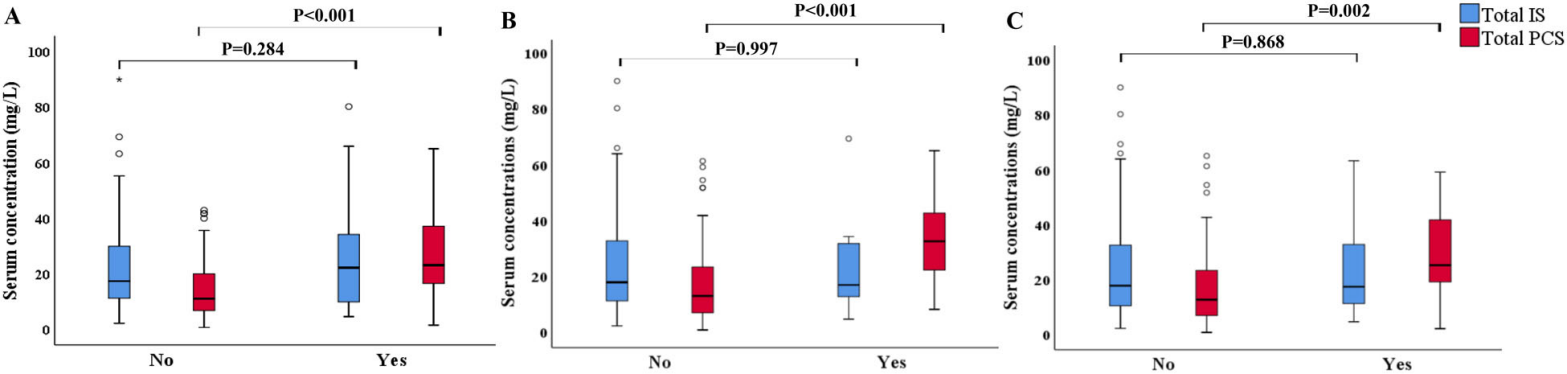
Serum concentration of total-PCS and total-IS in PD patients with different endpoints. Distribution of (A) PD failure event, (B) cardiovascular event, and (C) PD-associated peritonitis.

During the study period (mean follow-up period: 51.3 months; median: 60; range: 46–60), 34 (26.8%) patients experienced PD failure event, among whom 26 transferred to HD due to inadequate dialysis or recurrent peritonitis. Eight patients (6.3%) died during follow-up. In addition, 17 (13.4%) patients experienced PD-associated peritonitis, and 10 (7.9%) had newly occurred cardiovascular event. The distribution of total PCS and IS levels by different endpoints was shown in Figure 1, total PCS were higher in patients who experienced PD failure event, cardiovascular event, PD-association peritonitis (*p* < 0.001, *p* < 0.001, *p* = 0.002, respectively) than those without.

#### Serum PCS was a risk factor for clinical outcomes in PD

Multivariate cox regression analysis revealed that total PCS (HR: 1.05, 95% CI = 1.02 to 1.07, *p* < 0.001), age (HR: 1.03, 95% CI = 1.00 − 1.05, *p* = 0.031), and peritoneal Kt/V (HR: 1.35, 95% CI = 1.05 to 1.73, *p* = 0.019) were independently associated with PD failure event, as showed in Table 2. It seemed that higher total PCS was a detrimental risk factor, while residual renal Kt/V and peritoneal Kt/V did not contribute to less PD failure in the multivariate cox regression analysis. Meanwhile, older age (HR: 1.10, 95% CI = 1.04 to 1.16, *p* = 0.001) and higher total PCS (HR: 1.08, 95% CI = 1.04 to 1.13, *p* < 0.001) increased the risk of having cardiovascular event. As for PD-association peritonitis, total PCS (HR: 1.04, 95% CI = 1.02 to 1.08, *p* = 0.001) was the only independent factor. The serum total and free IS was nonrelated to any clinical outcome in univariate and multivariate analyses. These results showed that total PCS played a critical role in clinical outcomes of PD patients.

**Table 2.** Univariate and multivariate cox regression analysis for evaluating the relationship between independent variables and clinical outcomes.

Variables	PD failure event				Cardiovascular event				PD-associated Peritonitis	
	Univariate cox regression		Multivariate cox regression		Univariate cox regression		Multivariate cox regression		Multivariate cox regression	
	HR (95% CI)	*p* value	HR (95% CI)	*p* value	HR (95% CI)	*p* value	HR (95% CI)	*p* value	HR (95% CI)	*p* value
Age (years)	1.03 (1.01-1.05)	0.017	1.03 (1.00-1.05)	0.031	1.07 (1.02-1.12)	0.007	1.10 (1.04-1.16)	0.001	0.97 (0.60-1.57)	NS
Male (%)	2.13 (0.99-4.58)	0.051	2.11 (0.96-4.64)	0.065	1.59 (0.41-6.15)	NS			0.99 (0.96-1.03)	NS
PD duration (months)	0.99 (0.91-1.09)	NS			0.99 (0.84-1.16)	NS			1.08 (0.84-1.16)	NS
MS (%)	1.04 (0.32-3.41)	NS			2.66 (0.57-12.53)	NS			0.65 (0.09-4.89)	NS
Hypertension (%)	0.76 (0.23-2.48)	NS			0.04 (0-257.51)	NS			0.04 (0-33.11)	NS
Diabetes mellitus (%)	0.54 (0.22-1.30)	NS			2.00 (0.43-9.43)	NS			0.47 (0.06-3.53)	NS
Total-IS (mg/L)	1.01 (0.99-1.03)	NS			1.00 (0.95-1.04)	NS			0.99 (0.97-1.03)	NS
Free-IS (mg/L)	0.90 (0.72-1.12)	NS			0.93 (0.64-1.35)	NS			0.93 (0.69-1.24)	NS
Total-PCS (mg/L)	1.04 (1.02-1.07)	<0.001	1.05 (1.02-1.07)	<0.001	1.06 (1.02-1.09)	0.001	1.08 (1.04-1.13)	<0.001	1.04 (1.02-1.08)	0.001
Free-PCS (mg/L)	1.14 (0.92-1.41)	NS			1.23 (0.85-1.78)	NS			1.01 (0.72-1.43)	NS
Peritoneal transport type (%)	0.58 (0.20-1.70)	NS			0.67 (0.07-5.32)	NS			0.95 (0.01-4.85)	NS
D/Pcr	9.26 (0.25-20.21)	NS			1.06 (0-924.35)	NS			0.03 (0.01-6.34)	NS
Peritoneal Kt/V	1.48 (1.16-1.89)	0.002	1.35 (1.05-1.73)	0.019	0.68 (0.19-2.46)	NS			0.67 (0.26-1.75)	NS
Residual renal Kt/V	0.52 (0.27-1.00)	0.050	0.82 (0.41-1.61)	0.555	0.91 (0.37-2.24)	NS			1.04 (0.56-1.93)	NS
Peritoneal Ccr	1.01 (0.98-1.03)	NS			0.95 (0.89-1.01)	NS			0.96 (0.92-1.01)	NS
Residual renal Ccr	1.00 (0.99-1.01)	NS			1.00 (0.98-1.02)	NS			1.00 (0.99-1.01)	NS
nPCR (g/kg/day)	0.14 (0.02-1.01)	0.051	0.18 (0.02-1.64)	0.107	0.63 (0.02- 18.40)	NS			0.85 (0.06-11.22)	NS
Hemoglobin (g/L)	0.99 (0.98-1.02)	NS			1.01 (0.98-1.05)	NS			0.99 (0.97-1.03)	NS
Albumin (g/L)	1.02 (0.95-1.09)	NS			1.03 (0.91-1.17)	NS			1.02 (0.92-1.12)	NS
Creatinine (µmol/L)	1.00 (0.99-1.01)	NS			1.00 (0.99-1.00)	NS			1.00 (0.99-1.01)	NS
Uric acid (µmol/L)	1.00 (0.99-1.01)	NS			1.00 (0.99-1.01)	NS			1.00 (0.99-1.00)	NS
Calcium (mmol/L)	1.40 (0.43-4.54)	NS			0.36 (0.05-2.77)	NS			0.41 (0.08-2.03)	NS
Phosphate (mmol/L)	0.74 (0.41-1.35)	NS			1.10 (0.39-3.10)	NS			1.43 (0.67-3.05)	NS
Bicarbonate (mmol/L)	0.98 (0.90-1.05)	NS			1.01 (0.87-1.17)	NS			1.01 (0.90-1.13)	NS
PTH (pg/mL)	1.00 (0.99-1.01)	NS			1.00 (0.99-1.01)	NS			0.99 (0.99-1.00)	NS

Ccr: creatinine clearance rate; CI: confidence interval; D/Pcr: Dialysate/plasma creatinine ratio; HR: hazard ratio; IS: indoxyl sulfate; MS: metabolic syndrome; PCS: *p*-cresol sulfate; PD: peritoneal dialysis; PTH: parathyroid hormone; nPCR: normalized protein catabolic rate.

#### Higher PCS levels predict worse prognosis in PD revealed by Kaplan-Meier curves

**Figure 2.**
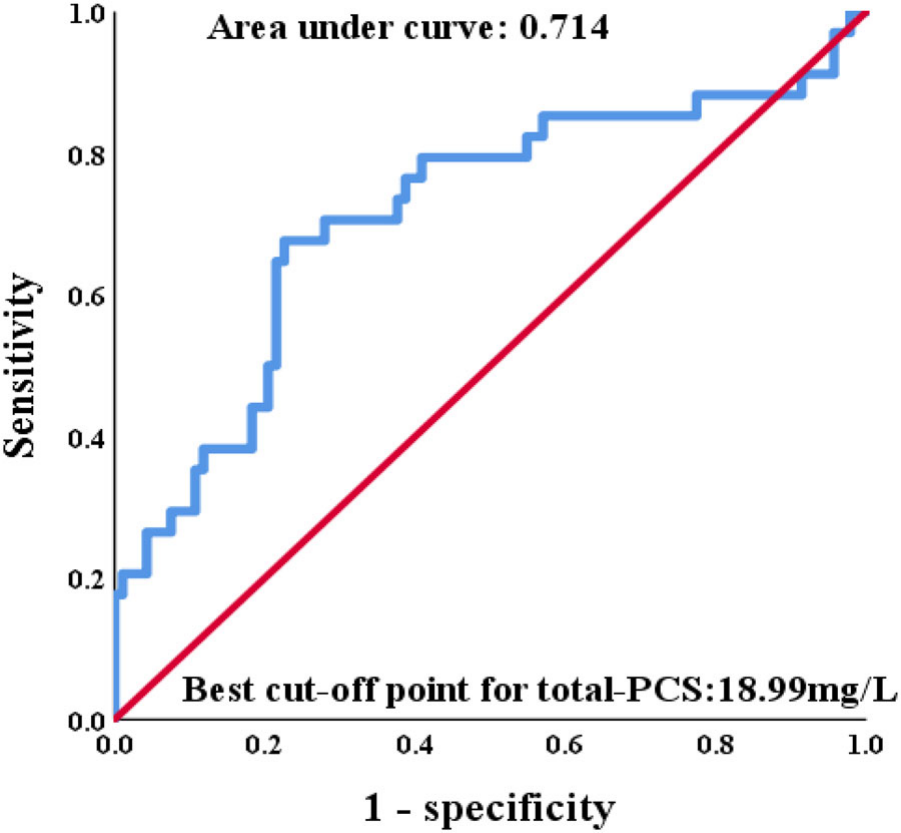
Receiver operator characteristic curve analysis for serum total-PCS level regarding 5-year PD failure event prediction among PD patients.

**Figure 3.**
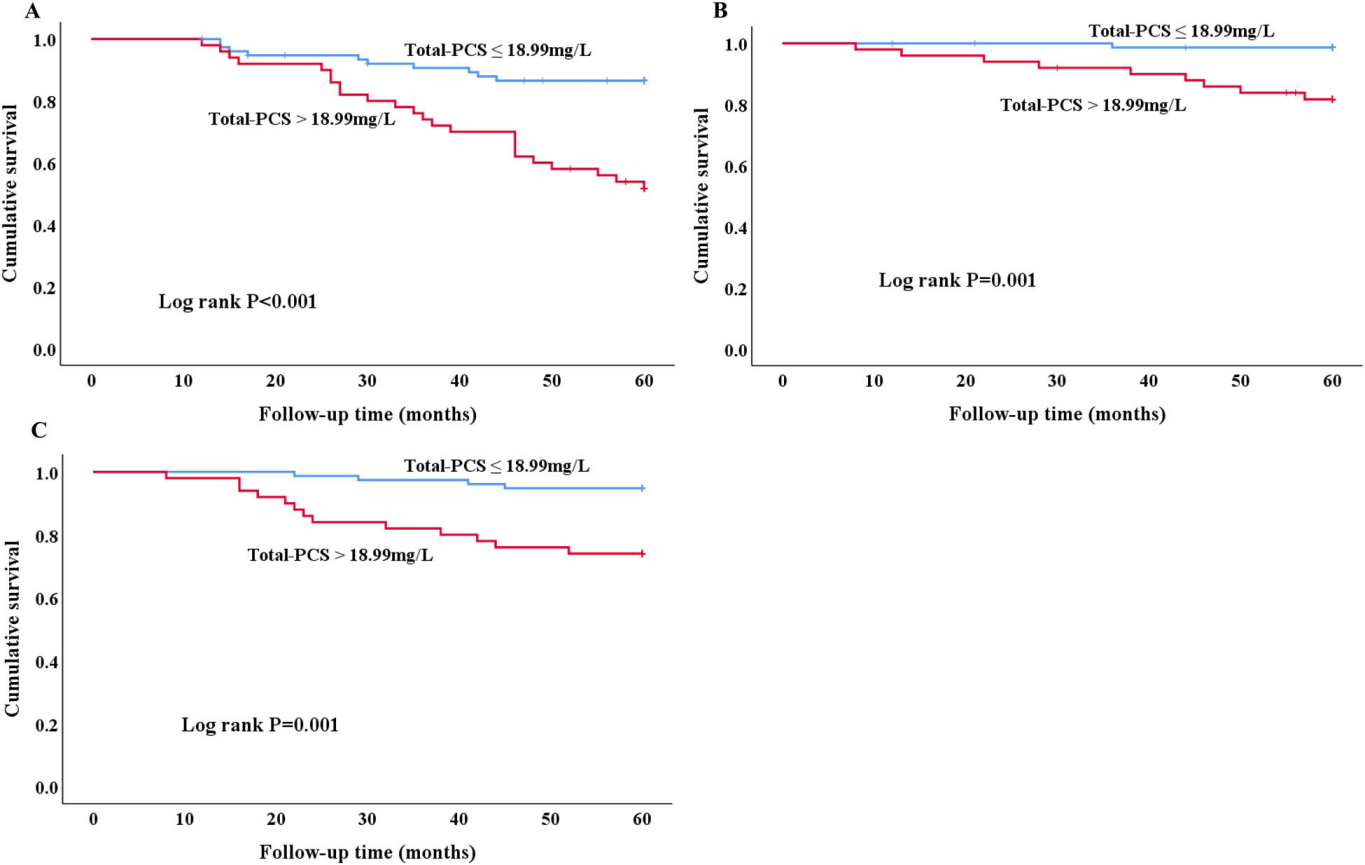
Kaplan-Merier curves of time to clinical endpoint. (A) PD failure event. Patients with high (>18.99 mg/L) total-PCS are compared to low (≤18.99 mg/L) total-PCS concentrations (log rank *p* < 0.001). (B) Cardiovascular event. Patients with high (>18.99 mg/L) total-PCS are compared to low (≤18.99 mg/L) total-PCS concentrations (log rank *p* = 0.001). (C) PD-associated Peritonitis. Patients with high (>18.99 mg/L) total-PCS are compared to low (≤18.99 mg/L) total-PCS concentrations (log rank *p* = 0.001).

A best cutoff point to evaluate the accuracy of predicting 5-year PD failure event from serum total PCS were obtained by ROC analysis (Figure 2). The area under curve from cutoff point analysis was 0.714. Divided by 18.99 mg/L based on the best cutoff value of total PCS (Figure 3), patients with lower total PCS demonstrated less PD failure event than those with higher total PCS (log-rank, *p* < 0.001). Moreover, patients with high total PCS levels had higher risk of cardiovascular event (log rank *p* = 0.001) and PD-association peritonitis (log rank *p* = 0.001) as well during 5-year follow-up.

### Correlated factors that influenced serum levels of PCS and IS

Total and free PCS and IS levels were interrelated (Table 3). Total IS negative co-related to residual renal function represented by Kt/V (*r* = −0.397, *p* = <0.001) and Ccr (*r* = −0.513, *p* = <0.001), while not for PCS. Meanwhile, total IS levels were positively co-related to peritoneal Kt/V (*r* = 0.240, *p* = 0.007) and Ccr (*r* = 0.207, *p* = 0.020), whereas not for PCS either. Other variables associated with total IS levels were diabetes mellitus, albumin, and creatinine.

**Table 3.** Correlations between Spearman rank of baseline characteristics with serum total-PCS and total-IS in PD patients.

Variables	Total-PCS		Total-IS	
	*r*	*p*	*r*	*p*
Age (years)	−0.085	0.395	−0.083	0.357
Male (%)	0.066	0.341	0.003	0.973
PD duration (months)	0.029	0.586	0.213	0.346
MS (%)	0.009	0.922	0.126	0.157
Hypertension (%)	0.073	0.415	0.114	0.203
Diabetes mellitus (%)	−0.089	0.319	0.178*	0.045
Total-PCS (mg/L)			0.198*	0.026
Free-PCS (mg/L)	0.635**	<0.001	0.331**	<0.001
Total-IS (mg/L)	0.198*	0.026		
Free-IS (mg/L)	0.046	0.608	0.666**	<0.001
Peritoneal transport type	−0.031	0.731	0.002	0.980
D/Pcr	−0.042	0.640	0.001	0.992
Peritoneal Kt/V	0.125	0.161	0.240**	0.007
Residual renal Kt/V	−0.030	0.742	−0.397**	<0.001
Peritoneal Ccr	−0.097	0.280	0.207**	0.020
Residual renal Ccr	0.027	0.766	−0.513**	<0.001
nPCR (g/kg/day)	0.094	0.295	−0.104	0.246
Hemoglobin (g/L)	−0.149	0.093	0.067	0.455
Albumin (g/L)	0.068	0.449	0.188*	0.034
Creatinine (µmol/L)	0.063	0.481	0.316**	<0.001
Uric acid (µmol/L)	0.014	0.874	−0.088	0.323
Calcium (mmol/L)	−0.001	0.989	−0.072	0.424
Phosphate (mmol/L)	0.048	0.592	0.001	0.990
Bicarbonate (mmol/L)	0.063	0.592	−0.007	0.939
PTH (pg/mL)	0.075	0.405	0.119	0.184

Ccr: creatinine clearance rate; D/Pcr: dialysate/plasma creatinine ratio; IS: indoxyl sulfate; MS: metabolic syndrome; nPCR: normalized protein catabolic rate; PCS: *p*-cresol sulfate; PD: peritoneal dialysis; PTH: parathyroid hormone. r* indicates *p* value less than 0.05 and r** indicates *p* value less than 0.001.

### Meta analysis showed diverse role of PCS and IS on clinical outcomes at different stages of CKD

**Figure 4.**
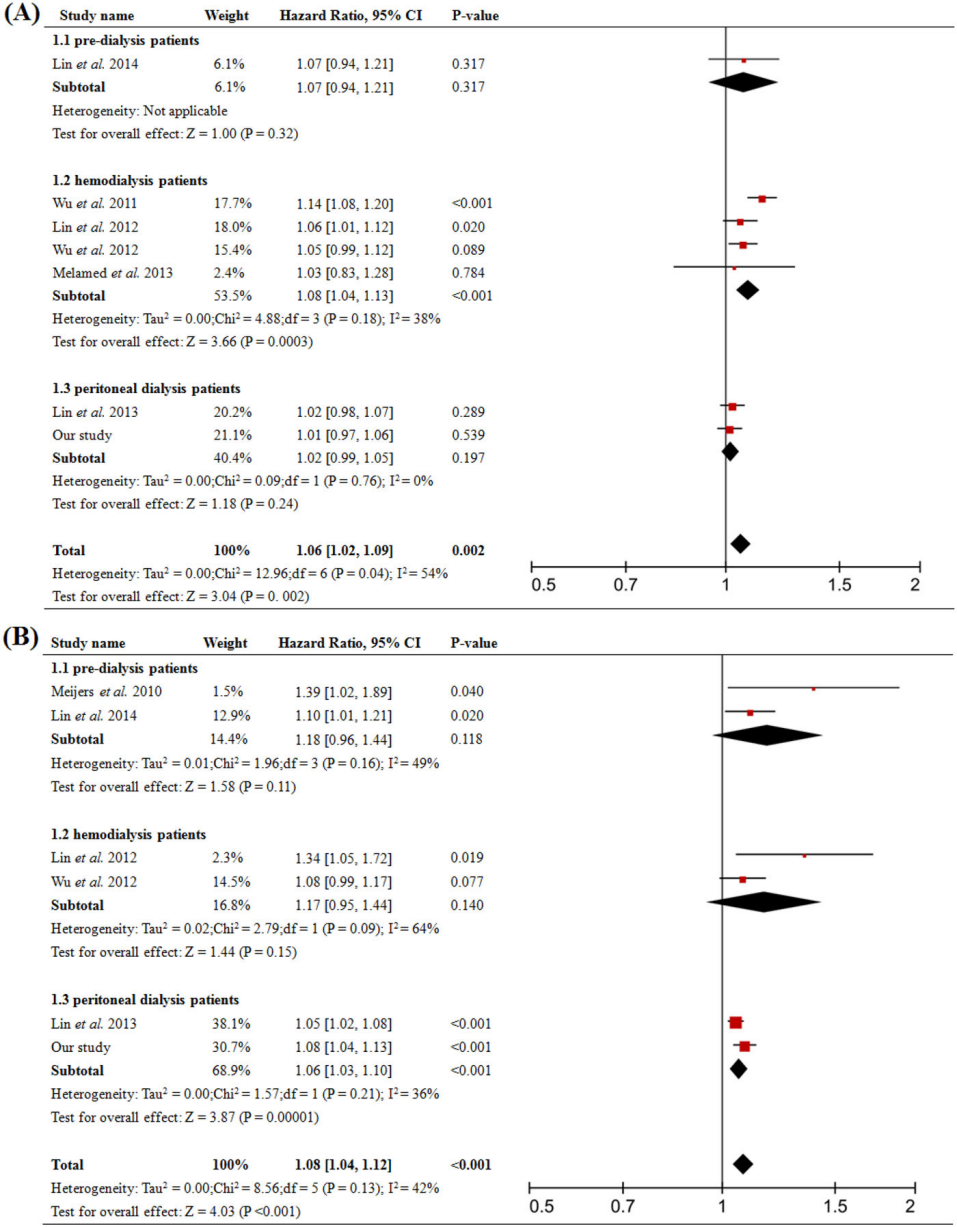
Forest plots of association between serum total-PCS and the risk of clinical outcome in in pre-dialysis and dialysis patients. (A) Total-PCS and all-cause mortality and (B) total-PCS and cardiovascular event. CI, confidence interval.

**Figure 5.**
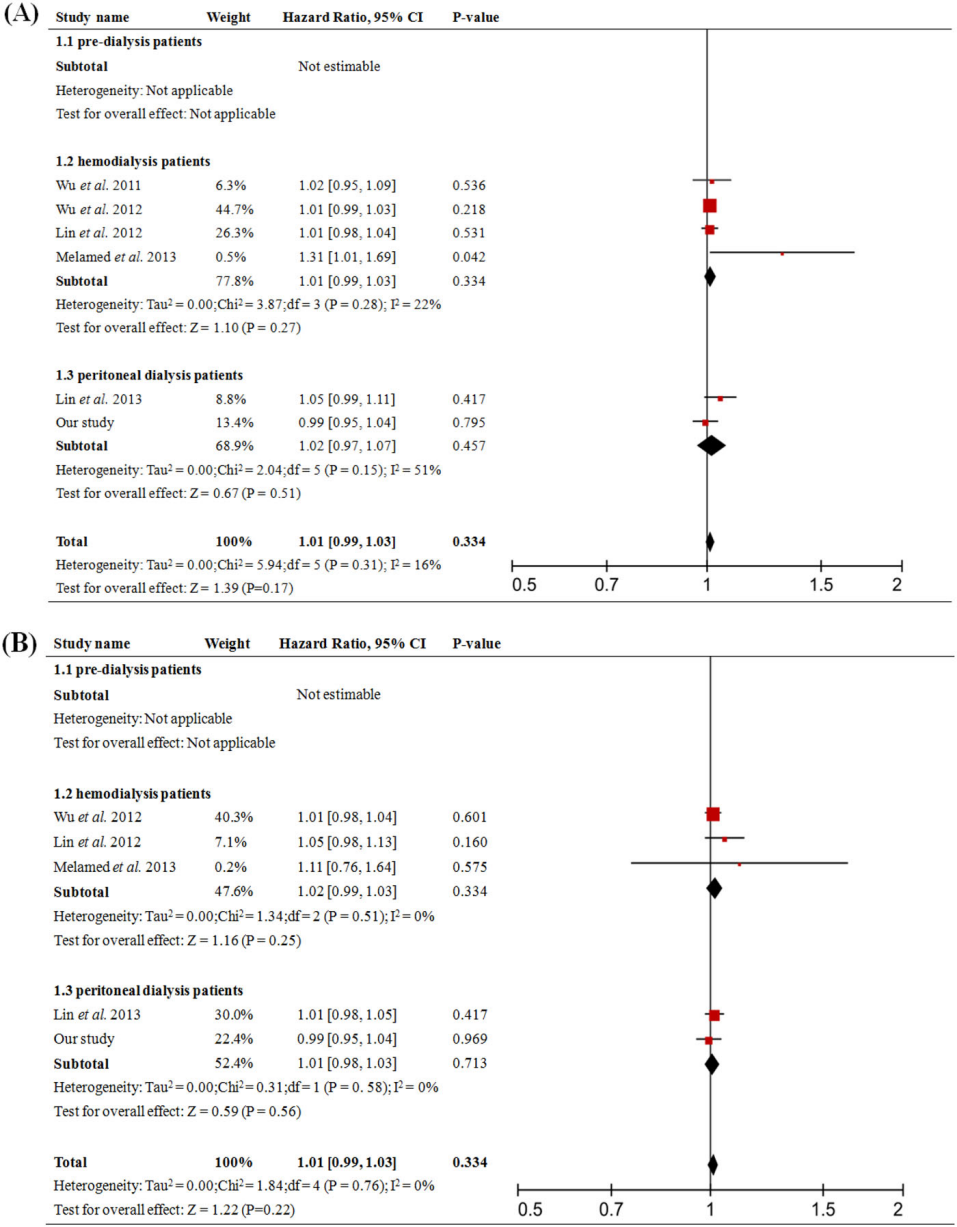
Forest plots of association between serum total-IS and the risk of clinical outcome in pre-dialysis and dialysis patients. (A) Total-IS and all-cause mortality, (B) Total-IS and cardiovascular event. CI, confidence interval.

A total of 11 prospective cohort studies were included and the baseline characteristics were shown in Table 4. The overall analysis showed that higher total PCS significantly increased risk of all-cause mortality (pooled HR = 1.06, 95% CI = 1.02 to 1.09, *p* = 0.002, Figure 4(A)) and cardiovascular event (pooled HR = 1.08, 95% CI = 1.04 to 1.12, *p* < 0.001, Figure 4(B)); while the analysis of total IS showed no significant difference (Figure 5). In the subgroups analysis, total PCS was associated with all-cause mortality (pooled HR = 1.08, 95% CI = 1.04 to 1.13, *p* < 0.001, Figure 4(A) 1.2) in HD patients but not in PD; it also increased the risk of cardiovascular event (pooled HR = 1.06, 95% CI = 1.03 to 1.10, *p* < 0.001, Figure 4(B) 1.3) in PD patients.

**Table 4.** Characteristics of studies in the CKD or dialysis patients using PCS or IS as a prognostic indicator.

Author (Year)	Type of Patients	Number	Age (y)	Male (%)	Follow-up time	Total-PCS (mg/L)	Free-PCS (mg/L)	Total-IS (mg/L)	Free-IS (mg/L)
Bammens *et al.* 2006 [25]	Stage 5 CKD	175	64.7 ± 1.1	61.7	30.1 ± 0.4 mo	19.0 ± 0.9	2.59 ± 0.17	NR	NR
Meijers *et al.* 2010 [9]	Stage 1-5 CKD	499	64 (50-75)	55.0	33 mo	43.6 (17.8, 91.3)	1.57 (0.60, 3.56)	NR	NR
Liabeuf *et al.* 2010 [8]	Stage 1-5 CKD	150	67 ± 12	60.0	779 ± 187 d	18.9 ± 17.3	2.6 ± 5.1	NR	NR
Wu *et al .*2011 [11]	HD	268	66.9 ± 12	57.5	21 ± 3 mo	7.16 (<1, 42.06)	NR	4.63 (<0.225, 53.58)	NR
Wu *et al.* 2012 [24]	HD	112	72.6 ± 6.3	30.4	33.2 mo	28.2 (1, 98.7)	2.2 (0.01, 25.6)	35.0 (0.23, 84.5)	3.7 (0.01, 19.7)
Lin *et al.* 2012 [12]	HD	50	70.5 ± 3.45	NR	38 mo	21.99 ± 12.08	1.59 ± 1.12	40.54 ± 16.73	4.27 ± 2.90
Chen *et al.* 2012 [5]	HD	91	57.6 ± 1.2	42.9	3.5 y	25.4 ± 1.9	2.1 ± 0.2	36.2 ± 1.7	2.9 ± 0.2
Lin *et al.* 2013 [7]	Peritoneal dialyis	46	47.4 ± 12.8	45.6	5 y	17.4 ± 14.6	1.1 ± 1.0	40.9 ± 15.7	4.3 ± 2.6
Melamed *et al.* 2013 [13]	HD	521	58.3 ± 14.7	54.1	NR	NR	NR	NR	NR
Lin *et al.* 2014 [10]	Stage 3-5 CKD	72	60.1 ± 9.4	50.0	3 y	7.7 ± 7.2	NR	NR	NR
Our study	Peritoneal dialyis	127	44.7 ± 13.9	60.6	5 y	17.8 (7.2, 25.2)	1.44 (0.51, 1.79)	23.6 (11.1, 32.6)	1.90 (0.65, 2.18)

CKD: chronic kidney disease; NR: not reported; PCS: *p*-cresol sulfate.

## Discussion

The clinical significance of PBUTs in PD patients needs further investigation. Our study showed that serum total PCS, one of major types of PBUTs, not only contributed to the occurrence of PD failure event, but also increased the risk of cardiovascular event; whereas, it did not contributed to higher all-cause mortality in PD. These observations were similar to the findings reported by Lin et al. [7]. In addition, we also found that patients with higher total PCS had higher rate of PD-associated peritonitis. PCS was one of the metabolized products of intestine bacteria. It has been proved that sustained dialysis could lead to numerous alteration in the gut microbiome featured with disproportion among guarding and pathogenic microbiota and ectopic intestinal flora [1,2,19]. Recent studies have also shown that gut dysbiosis could induce accumulation of systemic inflammation uremic toxins and infections, which might explain the relevance between serum total PCS levels and PD-associated peritonitis [19]. The causal linkage with gut microbiome deserves further investigation.

We did the further meta-analysis to explore the influence of PCS and IS on prognosis of different stages of CKD, including pre-dialysis CKD, HD, and PD patients. The overall effect of elevated serum total PCS was significantly associated with higher risk of cardiovascular event and all-cause mortality including dialysis and nondialysis subjects. Intriguingly, subgroups analysis in patients undertaking PD revealed that total PCS was responsible for the cardiovascular event, but not for all-cause mortality (Figure 4), which was consistent with what we discovered with our cohort. Besides, total PCS was related to an increased risk of all-cause mortality in HD patients, but not cardiovascular event. While in pre-dialysis CKD patients, total PCS was not predictive of either cardiovascular event or all-cause mortality. Moreover, serum total IS level did not influence cardiovascular event and all-cause mortality in both overall and subgroup analysis (Figure 5). Results from experimental studies revealed that PCS could cause endothelial damage by suppressing the activity or activated leucocytes, promoting endothelial microparticle release, and disrupting the adherences junctions [2,3,20]. PCS may also cause endothelial and vascular dysfunction through promoting vascular smooth muscle cells proliferation, which is one of the major factors contributing to intima media thickness. All these might explain the underlying mechanism between elevation of total PCS levels and increased risk of death due to cardiovascular diseases. Furthermore, our analysis showed that divided by the best cutoff value from the ROC curve, patients with total PCS higher than 18.99 mg/L had worse prognosis. Higher PCS not only increased the risk of worse outcomes, but also predicted patients’ long-term prognosis in PD. Future studies with larger sample sizes are required to explore the reliability and effectiveness of this index in PD.

Considering the diverse effect of serum PCS and IS on patients’ prognosis in PD, we analyzed the potent influential factors of the two major types of PBUTs (Table 3). We observed a negative correlation between total IS and residual renal Kt/V at baseline, which was similar to the previous studies in nondialytic CKD [21]. Barreto et al. [21] discovered that there was a gradual and progressive increase in serum IS with increasing CKD stages. It was interesting that IS also positively correlated to peritoneal Kt/V and Ccr as well as baseline serum creatinine in our study. Considering its weak influence on patients’ outcomes in our study on PD, the meaning of its connection with creatinine clearance needs future discussion. Meanwhile, we did not found significant correlation between PCS and renal creatinine clearance, which was also observed by van Gelder et al. in HD [22]. This indicated that the prognostic value of PCS on PD was independent of small-molecular-weight toxin metabolism. Moreover, the PCS and IS levels were weakly bidirectional interrelated, which might be due to overlap between their metabolic pathway. PCS and IS both derive from microbial protein fermentation in the intestine by degrading tryptophan and tyrosine after absorption, which were ultimately metabolized to generate PCS and IS [2,3]. Studies in healthy individuals showed that the production of *p*-cresol or indoxyl can be increased or decreased by dietary protein, carbohydrates, and fiber [20,23,24]; however, we could not determine differences in protein dietary intake and function of the gut microbiome that may partly account for absence of a direct relationship between PCS and residual renal function in our study. Meanwhile, the role of albumin is complex as a main carrier protein. PCS and IS circulates noncovalently bound to albumin and competes for one high-affinity albumin-binding sites (Sudlow site II) which might lead to their different clearance [6,25]. Further studies are needed to elucidate the different metabolic pathway, pathophysiologic role, degree, affinity of protein binding, and dialytic clearance of the PCS and IS.

In recent years, therapeutic strategies have also been developed aiming at lowering PBUTs, mainly represented by the serum concentrations of PCS and IS in CKD patients to observe their beneficial effects, including prebiotic [26–28], Curcumin [29], Shen-Shuai-Ning [17], and Sevelamer [30,31]. Our group previously conducted a case-control study investigating the effect of Shen-Shuai-Ning granule on the serum PCS and IS concentrations in 60 PD patients. After 12 weeks treatment of Shen-Shuai-Ning granule, there was a significant reduction in total PCS [17]. Similar result was observed by Ramos et al. [32] in subjects with 50 CKD patients who received Prebiotic for three months and by Meijers et al. [27] in individuals with HD who received prebiotic oligofructose-enriched inulin for four weeks. The symbiotic may decrease ammonium hydroxide production from ammonia, which decrease the intestinal luminal pH, preventing dysbiosis and intestinal mucosa alteration and, decreasing uremic toxins production and systemic inflammation [19,33]. However, the beneficial effects of these medications on patients’ survival and clinical outcomes among pre-dialysis and dialysis patients remain unclear. Future well designed multicenter randomized clinical trials are urged to clarify the benefit and mechanism of reducing PCS or IS to improve prognosis in CKD population, especially PD patients.

The major limitation of the study presented here is that all subjects in our study were enrolled from one medical center and sample bias existed due to its nature. The statistical power was limited because of the small sample size. Moreover, some of the concomitant medications changed frequently in the PD patients during the follow-up when they came to the out-patient department; thus we did not include them as parameters in the prognosis analysis; however, they might be the influential factors for serum PCS and IS levels, which we will pay more attention to in our future study design.

## Conclusion

The serum total PCS concentration was a detrimental factor for higher dialysis failure event, cardiovascular event, and PD-associated peritonitis in sustained PD patients. It could be used as an innovative marker in predicting poor clinical outcomes in PD. Further studies are necessary to investigate the relationship between therapeutic methods of lowering the serum PCS levels and improving clinical outcomes of pre-dialysis CKD and dialysis patients.
